# Influence of the Human Development Index on the Maternal–Perinatal Morbidity and Mortality of Pregnant Women with SARS-CoV-2 Infection: Importance for Personalized Medical Care

**DOI:** 10.3390/jcm10163631

**Published:** 2021-08-17

**Authors:** Yolanda Cuñarro-López, Santiago García-Tizón Larroca, Pilar Pintado-Recarte, Concepción Hernández-Martín, Pilar Prats-Rodríguez, Óscar Cano-Valderrama, Ignacio Cueto-Hernández, Javier Ruiz-Labarta, María del Mar Muñoz-Chápuli, Óscar Martínez-Pérez, Miguel A. Ortega, Juan Antonio De León-Luis

**Affiliations:** 1 Department of Public and Maternal and Child Health, School of Medicine, Complutense University of Madrid, 28040 Madrid, Spain; ycunarro@ucm.es (Y.C.-L.); gineteca@gmail.com (S.G.-T.L.); ppintadorec@yahoo.es (P.P.-R.); chermartin@telefonica.net (C.H.-M.); ignaciocuetohernandez@gmail.com (I.C.-H.); javruila@hotmail.com (J.R.-L.); mar.mchg@gmail.com (M.d.M.M.-C.); jaleon@ucm.es (J.A.D.L.-L.); 2 Department of Obstetrics and Gynecology, University Hospital Gregorio Marañón, 28009 Madrid, Spain; 3 Health Research Institute Gregorio Marañón, 28009 Madrid, Spain; oscarcanovalderrama@hotmail.com; 4 Department of Gynecology and Obstetrics, QuirónSalud Dexeus University Hospital, 08028 Barcelona, Spain; pilpra@dexeus.com; 5 Obstetrics and Gynaecology Department, Puerta de Hierro University Hospital, 28222 Madrid, Spain; 6 Unit of Histology and Pathology, Department of Medicine and Medical Specialities, Faculty of Medicine and Health Sciences, University of Alcalá, 28801 Alcalá de Henares, Spain; miguel.angel.ortega92@gmail.com; 7 Ramón y Cajal Institute of Sanitary Research (IRYCIS), 28034 Madrid, Spain; 8 Cancer Registry and Pathology Department, Hospital Universitario Principe de Asturias, 28806 Madrid, Spain

**Keywords:** coronavirus disease-19 (COVID-19), pregnancy, human development index (HDI), maternal–perinatal outcomes, maternal–perinatal morbidity and mortality

## Abstract

Coronavirus disease-19 (COVID-19) is perhaps the most worrisome pandemic in the 21st century, having entailed devastating consequences for the whole society during the last year. Different studies have displayed an existing association between pregnancy and COVID-19 severity due to the various physiological changes that occur during gestation. Recent data identified maternal country of origin as an important determinant of COVID-19 presentation in pregnant women. However, the explanation of this fact remains to be fully elucidated. Therefore, the purpose of this work is to analyze the possible relationship between Human Development Index (HDI) of maternal country of origin with the morbimortality of pregnant women and their newborns. Here, we conducted a multicentric, ambispective, observational case-control study (1:1 ratio) and compare with the HDI of each country (group 1—very high HDI, group 2—high HDI, group 3—medium HDI, and group 4—low HDI). In total, 1347 pregnant women with confirmed SARV-CoV-2 infection (cases) were enrolled, and each was paired with one control to give a total number of 2694 participants from 81 tertiary care centers. Among the women with SARS-CoV-2 infection, more cases were produced of perinatal mortality, overall maternal morbidity, COVID-19 maternal morbidity, C-sections, hypertensive maternal morbidity, and perinatal morbidity. Our results described an inverse association between HDI and maternofetal morbidity and mortality. Moreover, the countries with an HDI lower than 1 showed higher rates of patients with maternal COVID-19-related morbidity (6.0% vs. 2.4%, *p* < 0.001), a need for oxygen therapy (4.7% vs. 1.8%, *p* < 0.001), and maternal ICU admission (2.6% vs. 1.0%, *p* = 0.007). Compared to other risk factors such as overweight, obesity, preexisting and obstetric comorbidities, HDI emerged as an independent risk factor explaining much of the increased maternal–perinatal morbidity and mortality detected in our group of cases. Further research is needed to establish to confirm the real impact of this factor and its components on pregnancy outcomes.

## 1. Introduction

The global pandemic caused by severe acute respiratory syndrome coronavirus 2 (SARS-CoV-2), responsible for coronavirus disease 2019 (COVID-19), has been growing at an accelerating rate. At the time of writing (22 February 2021), there have been more than 111,000,000 confirmed cases of COVID-19, and more than 2,463,000 associated deaths reported to the World Health Organization [1].

Pregnant women may be susceptible to developing more severe symptoms after infection with respiratory viruses due to physiological changes in the immune and cardiopulmonary systems that occur during pregnancy [2]. As in non-pregnant patients, most obstetrics patients with SARS-CoV-2 infection show only mild or no symptoms [3]. However, particular attention should be paid to pregnant women with an underlying disease, as they carry a higher risk of developing severe disease than the general population [4]. Most mothers with confirmed COVID-19 are discharged without any major complications, especially if they are asymptomatic [5]. However, this subset of patients shows a greater risk of maternal–perinatal morbidity and mortality (MPMM) such as a higher risk of C-section, premature birth [6], mechanical ventilation, maternal Intensive Care Unit (ICU) admission [7], low birth weight, Neonatal Intensive Care Unit (NICU) admission, and perinatal mortality [8]. To jointly assess MPMM events, we defined the concept of risk of loss of maternal and perinatal well-being. This concept in clinically important as it is a single event with impacts on both mother and newborn as the situation could arise of a maternal or perinatal death associated or not with the morbidity–mortality of either counterpart.

In the last months of this pandemic, correlations between several risk factors and maternal–perinatal morbidity–mortality events have been assessed in patients with COVID-19, such as maternal age above 35 years, overweight, obesity [9], preexisting maternal comorbidities such as diabetes or asthma [10], or obstetric morbidities [11]. In a review of the profiles of pregnant women acquiring SARS-CoV-2 infection during the first wave of the pandemic according to country of publication, we concluded that differences in maternal characteristics probably exist depending on the country they come from [12].

In the present study, we sought to determine if these differences in the profile of a pregnant woman with COVID-19 could be correlated with the inherent characteristics of the patients themselves, the present state of knowledge about the pandemic or with other biopsychosocial factors such as the Human Development Index (HDI), which is reported yearly by the United Nations Development Program (UNDP) [13]. The HDI is a summary measure of average achievement in key dimensions of human development: a long and healthy life, being knowledgeable, and have a decent standard of living. The HDI is the geometric mean of normalized indices for each of the three dimensions [13].

Our interest in examining possible associations between the socioeconomic factors encompassed by the HDI and the incidence of COVID-19 and its related morbidity and mortality was prompted by studies such as those by Shahbazi et al. [14] and Liu et al. [15], suggesting that population from countries with a higher HDI will have a greater risk of COVID-19 infection and mortality.

However, none of these studies has provided information about a possible link between the HDI of maternal country of origin and the morbidity–mortality of pregnant women and their newborns. With the hypothesis in mind that each country is likely to have its own idiosyncrasies, the present study was designed to examine the relationship between maternal and perinatal outcome events in patients with an active SARS-CoV-2 infection according to the HDI of their country of origin.

## 2. Materials and Methods

### 2.1. Study Design

This multicentric, ambispective, observational case-control study was performed according to Strengthening the Reporting of Observational Studies in Epidemiology (STROBE) guidelines [16].

Participants were obstetric patients registered by the Spanish Obstetrics Emergency group at various public and private Spanish hospitals. This national registry was set up in March 2020 to address the need to know the morbidity that SARS-CoV-2 infection produces in the mother–newborn binomial as well as monitor the interventions and measures needed to improve the care given to these patients [17].

Data collection was performed with a standard form, and the data were entered by the head researcher at each center after childbirth. Follow-up duration postpartum was 6 weeks.

The diagnosis of SARS-CoV2 infection was made by detecting the RNA of the virus through real-time reverse transcription polymerase chain reaction (RT-PCR) on a nasal swab, sputum, or throat swab [6].

The study period was 1 March 2020, during the first wave of SARS-CoV-2 infection in Spain, to 3 November 2020.

Cases were patients with confirmed active SARS-CoV-2 infection during their pregnancy and delivery. The control group was made up of pregnant women managed at the same centers during their pregnancy and labor who tested negative in a SARS-CoV-2 RT-PCR test.

The case/control ratio was 1:1. Cases were paired with controls according to delivery time such that each center selected SARS-CoV-2-negative asymptomatic patients who gave birth immediately before or after each infected mother did so. This method was implemented to correct for center conditions and management at the time of delivery and to decrease the risk of selection bias.

Patients were excluded if 6 weeks of peripartum follow up could not be completed.

The Human Development Index (HDI) is a summary measure of a country’s average level of achievement in the following major dimensions of human development: living a long and healthy life, being educated, and having a decent standard of living. Life expectancy serves as an indicator of the health dimension; standard of living is measured in terms of gross national income per capita; and education level is estimated as the average number of years of schooling among adults aged 25 years and older and expected number of years of schooling among children [13]. A country is given a higher HDI when its population has a higher life expectancy, education level, and gross national income per capita. One of the positive aspects of the HDI is that it is useful to compare countries with a higher rent per capita with other countries with a lower socioeconomic status but with a better educational or sanitary level.

These HDIs are reported within the annual Human Development Report published by the United Nations Development Program (UNDP) [13]. The UNDP divides countries into four broad categories of human development: group 1 (very high HDI), group 2 (high HDI), group 3 (medium HDI), and group 4 (low HDI) based on the numerical score obtained ranging from a minimum of 0 to a maximum of 1 [18]. For the present paper, we used HDIs published by the UNDP in 2020 corresponding to the year 2019 [19].

The variables collected in each participant were as follows: active SARS-CoV-2 infection status, presence of COVID-19 symptoms, country of origin, HDI and HDI group; maternal characteristics such as age, tobacco use, body mass index (BMI) (kg/m^2^), morbidities, nulliparous rate, COVID-19 symptoms at triage, and pneumonia rate; obstetrics and perinatal characteristics such as neonate morbidities, gestational age (GA) at triage, GA at delivery and birth weight; maternal and perinatal mortality data such as overall maternal morbidity including COVID-19 maternal morbidity (defined as a need for oxygen therapy when oxygen saturation of room air at rest is <94%), need for mechanical ventilation and maternal admission to an ICU, COVID-19-unrelated maternal morbidity such as C-section rate, maternal hemorrhagic disorder (abruptio placentae, accreta/increta/percreta placenta, postpartum hemorrhage or ruptured uterus) and hypertensive disorder (hypertension, preeclampsia, eclampsia, hypertensive encephalopathy or HELLP syndrome) [20]; and perinatal morbidity data such as GA at birth less than 37 weeks, neonatal near miss (defined as GA at birth less than 33 weeks, birth weight lower than 1750 g or Apgar score at 5th < 7 [21]) and admission to the NICU.

According to the seventh version of guidelines on the Diagnosis and Treatment of COVID-19 issued by the National Health Commission of China [22], COVID-19 severity was classified as follows:

1. Mild—clinical symptoms mild and no sign of pneumonia on imaging.
2. Moderate—fever and respiratory symptoms plus radiological findings of pneumonia.
3. Severe—any of the following conditions:
  - Respiratory distress (respiratory rate of ≥30 per min).
  - Oxygen saturation in room air at rest ≤93%.
  - Partial pressure of oxygen in arterial blood/fraction of inspired oxygen ≤300 mmHg.
4. Critical cases—any of the following conditions:
  - Respiratory failure and need for mechanical ventilation.
  - Shock.
  - Patients with failure of an organ requiring ICU care.

A descriptive study of all the patients included in the study was performed, and maternal, obstetric, and perinatal characteristics and MPMM were compared between cases and controls.

To jointly assess MPMM, we defined the concept of risk of loss of maternal–perinatal well-being. This concept is clinically important as it considers situations in which mortality and morbidity overlap during pregnancy management, especially at birth and in the postpartum period. It is a single event with impacts on both mother and newborn as the situation could arise of a maternal or perinatal death associated or not with the morbidity–mortality of either counterpart.

To analyze the risk of loss of maternal–perinatal well-being, the different maternal and perinatal morbidity events were graphically represented along with mortality and morbidity interrelations between mother and newborn both in cases and controls.

Next, we graphically represented the distribution of the patients in our series according to country of maternal origin, excluding Spanish patients, using a graded color intensity scale according to numbers of affected patients in each country.

The relationship between country of maternal origin HDI and mortality and morbidity events in the mother and newborn was first assessed irrespective of cases and controls in a quantitative manner through logistic regression analysis using the HDI score of each country.

Next, a qualitative analysis was conducted to assess the measure in which belonging or not to the same HDI group as Spain (group 1 or very high HDI) versus the rest of the HDI groups (groups 2–4, high, medium, and low HDI) was related to the different MPMM events.

Finally, to examine the associations between the two HDI categories and the MPMM events proving significant in the univariate analysis, an explanatory multivariate logistic regression analysis was carried out.

Possible effect-modifying or confounding variables were examined based on scientific knowledge and on clinically relevant factors such as maternal obesity and overweight, preexisting maternal comorbidities, and pregnancy-related morbidities [10,23,24].

### 2.2. Data Analysis

A specific database was designed to record the information necessary for this study. Statistical analysis was performed with the package Stata 13.1 (StataCorp LLC, College Station, TX, USA). Significance was set at *p* < 0.05. Quantitative variables were expressed as the mean (interquartile range or 95% confidence interval (CI)) and categorical variables as patient numbers and rates (%). For univariate analysis, Fisher’s exact test, chi-squared test or Student’s *t*-test, were used as appropriate.

The possible relationship between HDI group and maternal–perinatal morbidity was analyzed using a logistic regression model. Variables that were statistically or clinically (odds ratio (OR) > 1.5, OR < 0.67, Pearson correlation coefficient >0.1 or Pearson correlation coefficient <0.1) associated with HDI group and maternal-perinatal morbidity were included as possible confounding variables. Models indicating a change of less than 10% of the OR were assessed as candidates for the final regression model.

### 2.3. Ethical Approval

The authors declare that they have no conflict of interest. All procedures involving human participants were conducted in accordance with the ethical standards of the institutional and/or national research committee and were in line with the 1964 Helsinki declaration and its later amendments or comparable ethical standards. The registry’s objective updates were approved by the coordinating hospital’s Medical Ethics Committee on 23 March 2020 (reference number: PI 55/20) and each collaborating center subsequently obtained protocol approval locally. The registry protocol is available at ClinicalTrials.gov, identifier NCT04558996. For inclusion in the registry, patients were required to sign an informed consent form. When consent could not be obtained because of the urgency of the case or lack of protective equipment against the suspected infection, verbal consent was given, and this was noted in the patient’s medical record.

## 3. Results

**Figure 1:**
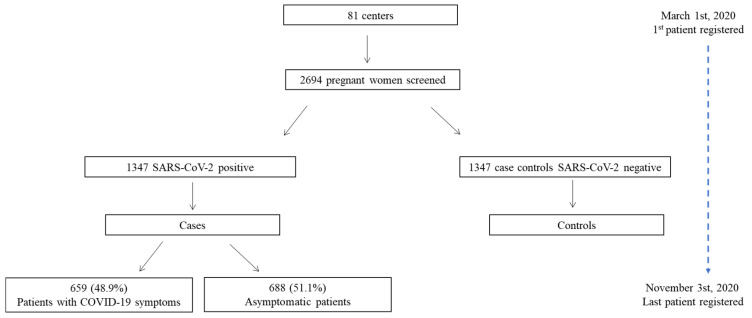
Flowchart showing the enrollment of cases and controls in our multicenter study and proportions of cases showing symptoms during the study period.

Over the period 1 March 2020 to 3 November 2020, 1347 pregnant women with confirmed SARV-CoV-2 infection (cases) were enrolled, and each was paired with one control to give a total number of 2694 participants from 81 tertiary care centers. Figure 1 shows the recruitment procedure including the number of cases showing COVID-19 symptoms.

In Table 1, we provide the maternal, obstetrics, and perinatal characteristics and MPMM data compiled for all participants and for the cases and controls. As may be observed, significant differences in most of the variables considered emerged between the two study groups. We should highlight that only two deaths were recorded among the cases and none were recorded in the control group. In addition, among the women with SARS-CoV-2 infection, more cases were produced of perinatal mortality (1.2% vs. 0.4%, *p* < 0.001), overall maternal morbidity (41.3% vs. 34.8%, *p* = 0.003), COVID-19 maternal morbidity (6.9% vs. 0.2%, *p* < 0.001) and all its components, along with a greater risk of C-section (27.8% vs. 19.9%, *p* < 0.001), hypertensive maternal morbidity (3.6% vs. 1.7%, *p* = 0.006), and perinatal morbidity (19.0% vs. 9.7%, *p* < 0.001). From the patients in the control group, one of the patients required oxygen therapy and two were admitted to the ICU.

**Figure 2:**
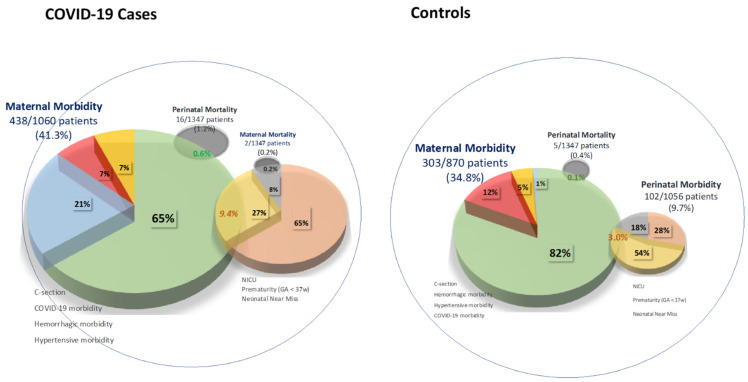
Maternal–perinatal morbidity and mortality and risk of loss of maternal–perinatal well-being by SARS-CoV-2 active infection status. In the figure, the proportionality of each entity over the whole number of cases and controls is conserved.

To better understand these differences, in Figure 2, the risk of loss of maternal–perinatal well-being is presented to illustrate the distributions of the different MPMM events described in Table 1. In general terms, it may be observed that the most frequent maternal–perinatal morbidity events recorded in both study groups were C-section and NICU admission. In addition, in this figure, we describe the clinical situations of interrelations between maternal and perinatal events. For example, in the cases group, there was a six times greater likelihood of experiencing perinatal mortality when there was maternal morbidity (*p* < 0.001) and up to a three-fold greater chance of presenting a joint maternal–perinatal morbidity event (*p* < 0.001).

**Figure 3:**
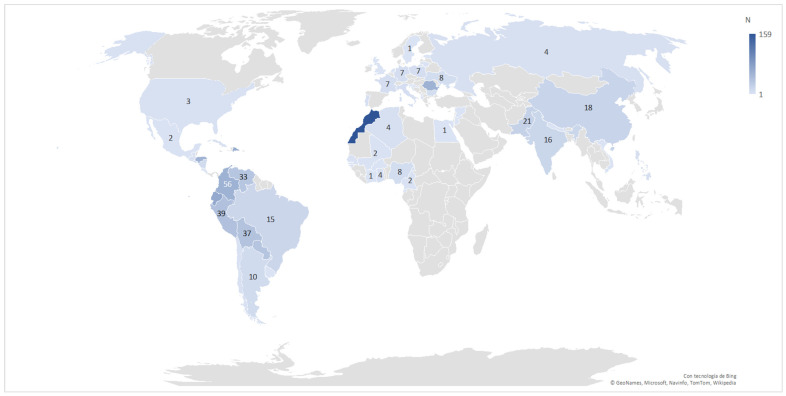
Cases of COVID-19 in pregnant women according to maternal country of origin.

Figure 3 showing maternal country of origin (excluding Spain) including 84% of all participants reveals that patients came from up to 59 different countries. The countries of origin of 1567 participants (69.2%) belonged to HDI group 1 (very high, including Spain) and those of 696 participants (30.8%) were in lower HDI categories than group 1: 389 women (17.2%) in group 2, 282 women (12.5%) in group 3, and 25 women (1.1%) in group 4. The country most represented by the participants after Spain was Morocco with 159 women (7.0%), followed by Ecuador with 66 (2.9%). Based on HDIs, it may be seen that Morocco belongs to group 3 with an HDI of 0.686 and Ecuador belongs to group 2 with an HDI of 0.759.

An inversely proportional trend was observed between HDI and the MPMM variables, although without attaining significance (OR = 0.51, 95% CI 0.21–1.23, *p* = 0.134).

The distribution of cases and controls according to MPMM variables is provided in Table 2. In 431 patients of the overall group (16.0%), the maternal country of origin was unknown. Of note, a significantly higher number of cases was recorded from countries with an HDI lower than 1 (65.5% high/medium/low HDI vs. 43.3% very high HDI, *p* < 0.001). In addition, this development index group showed higher rates of patients with maternal COVID-19-related morbidity (6.0% vs. 2.4%, *p*< 0.001), a need for oxygen therapy (4.7% vs. 1.8%, *p* < 0.001), and maternal ICU admission (2.6% vs. 1.0%, *p* = 0.007). No differences in the remaining variables examined were detected between the two HDI groups.

Our explanatory multivariate regression analysis revealed no modifying effect of the variables overweight and obesity or preexisting and obstetric maternal comorbidities on the relationship between maternal COVID-19 morbidity and HDI group (*p* = 0.545). Further, after correcting for confounding factors, the final model only included the qualitative variable HDI, such that coming from a country in HDI groups 2–4 was related to 2.6 times the likelihood of experiencing a maternal COVID-19 morbidity event (OR = 2.58, 95% CI 1.65–4.05, *p* < 0.001).

## 4. Discussion

A total of 1347 pregnant patients with confirmed active SARS-CoV-2 infection have been recruited from 80 centers during the first and second COVID-19 waves produced in Spain, and controls were 1347 pregnant women paired according to similar delivery dates returning a negative RT-PCR SARS-CoV-2 test result. Significant differences in MPMM outcomes were detected between our study cases and controls. Significant differences in country of origin HDI and maternal COVID-19 morbidity were also observed between cases and controls such that 820 of the pregnant women (36.2% of the participants) were from countries other than Spain. Our findings highlight the important role played by HDI compared with other cofactors in the morbidity of patients with SARS-CoV-2 during their pregnancy.

The present, along with two other series (617 pregnant women from 33 French maternal units [25] and 427 pregnant women from 194 UK obstetrics units [26]), is one of the larger series of reported cases to date. Our patients were also from a national cohort so that information was provided on the profile of pregnant women in our country. However, our recruitment period was much longer, and so our study offers improved knowledge of the physiopathology of this infection over the different waves produced of COVID-19.

Given the definition of our cases, we obviously detected a greater proportion of maternal symptoms compatible with COVID-19 and pneumonia in the case group (Table 1). Moreover, consistent with the findings of others, cases showed 5% more preexisting maternal comorbidities [10,27] and 6% more obstetric morbidities including preeclampsia [11]. In effect, pregnant women may be at an elevated risk of complications from SARS-CoV-2 infection possibly because SARS-CoV-2 enters the cell via the angiotensin-converting enzyme 2 (ACE2) receptor, which is upregulated in normal pregnancy leading to higher ACE2 expression [27].

Among the cases, maternal mortality was 0.2%, which is lower than the figure of 0.9% reported in the systematic review by Khalil et al. [28]. In this review, of note was the high maternal mortality recorded for Brazil of 36 out of 484 cases [29]. This country currently occupies the second position in the world in number of deaths due to COVID-19 [1].

We should stress that maternal mortality among our cases of SARS-CoV-2 infection was 42 times higher than the rate reported in the general population of pregnant women in Spain over the period 1999–2015 (148.5 vs. 3.57 deaths per 100,000 live births) [30], identifying COVID-19 as an important cause of maternal loss. Similarly, perinatal mortality was three times greater in the cases than controls (Table 1 and Figure 2), which is similar to the results provided by Khalil et al. [28]. Moreover, this increased risk was maintained when compared to the global perinatal mortality recorded in our country in 2019 [31]. Worsening of maternal health and the need for premature delivery of the fetus along with a lack of knowledge of the physiopathology of the disease during the first months of the pandemic could account for this increased mortality [12].

According to our maternal morbidity data, patients in the case group were up to 14 times more likely to be admitted to the ICU (2.7% vs. 0.2%, *p* < 0.001), which is in agreement with the 4% described by Allotey et al. [32]. The most likely reason was C-section in the cases (27.8% vs. 19.9%, *p* < 0.001), although overall, this figure is lower than that reported on the world scale [12]. Furthermore, while preterm births were twice as frequent in the cases than controls, their frequency was lower (11.1%) than the rate of around 22% reported in the literature [12,28]. A possible reason for these lower percentages was the longer recruitment period compared to that of prior studies ([12,28,32]). While not an objective of our study, it is likely that the different behavior of the pandemic and its management during the first and second waves could in part explain these results. More specifically, if we differentiate the cases of the first wave (ending late May 2020 in Spain) from the second wave (from August 2020 until the last recruitment date for this study), it may be seen that the first wave’s cases had a 30.1% rate of C-section and 12.4% preterm births versus 24.1% and 8.7%, respectively, recorded in the second wave, these differences being significant. The profile of the patients in the first and second waves of the COVID-19 pandemic in Spain has been previously analyzed by our group [33].

In our study, three women from the control group suffered a COVID-19 related maternal morbidity (two of them requiring admission in the ICU and one with oxygen therapy). Admission in the ICU could be explained because pregnant women without COVID-19 may require intensive care for obstetric or non-obstetric reasons [34]. The need of oxygen therapy in one patient without COVID-19 could be due to a respiratory disease not related with SARS-CoV-2 infection.

Our perinatal morbidity data indicate that the cases were 2.5 times more likely to undergo a near miss event and five times more likely to require NICU admission compared to controls, which was again determined by their prematurity and the presence of maternal infection, as many neonates are admitted for observation when mothers are diagnosed with COVID-19 [5].

As well as differentiating between maternal and perinatal outcomes, it is also useful to interrelate these findings (Figure 2). Cases showing maternal morbidity were significantly associated with an up to 3- and 6-fold greater risk of perinatal morbidity and mortality, respectively. This interrelated morbidity highlights the impacts of a mother’s health state on the morbidity–mortality of the fetus and newborn, whether associated with an increased risk of C-section or prematurity, determining the indivisible nature of the maternal-fetal binomial.

In our series, one in every three patients came from a country outside Spain, mostly Morocco. This figure is in agreement with the report by Larroca et al. [35] of a considerable rise in the last 15 years of foreign patients in Spain. To search for factors that could help identify pregnant women infected with SARS-CoV-2 with a greater risk of morbidity–mortality, we considered the variable HDI of the country of origin of these women. A non-significant inversely proportional trend was observed between the HDI of each country and MPMM. This contrasts with the findings of a study by Shahbazi et al. [14] who noted that countries with a greater HDI showed a higher incidence of morbidity and mortality due to COVID-19. This could be the consequence of an effective health-care system for early detection of disease, the implementation of screening programs to diagnose the disease in these countries, and the identification of asymptomatic and subclinical forms of disease.

The Human Development Index (HDI) is a measure described by the World Health Organization that includes the socioeconomic status of each country, the health level of the population, and the educational level. Therefore, the socioeconomic status is one of the components of the HDI [19]. According to the World Health Organization, social determinants of health explain the enormous inequality that exists in health when comparing some countries with others [36]. Considering that we live in a globalized world and that we are currently at a stage, according to many experts, fundamentally characterized by the mobility of people in search of new opportunities and a better quality life, the study of migratory flows and of the origin of people takes on special relevance. Women from developing countries represent more than 95% of all maternal deaths worldwide. Immigrants from middle- and low-income countries have a much higher risk of maternal death than those from the same origin living in high-income countries [37,38]. Moreover, in Western European countries, immigrant women have twice the risk of dying from complications of pregnancy and childbirth than native pregnant women [39]. It seems that the risk of maternal morbidity and mortality of these immigrants is reduced compared to what it would be in their places of origin, which can be explained by several factors, such as the better health conditions of women who have moved and their incorporation into a health system that provides higher quality health care during pregnancy [38]. Other socioeconomic factors could also have an influence on maternal morbidity, but it is clear that the country of the maternal origin is one of them.

When grouping the HDI data, it can be observed that the proportion of patients from countries with an HDI lower than that corresponding to group 1 was significantly higher in the cases than controls (Table 2). In addition, maternal morbidity, a need for oxygen therapy, and a need for maternal ICU admission were significantly higher in the patients from countries with an HDI lower than for group 1. Furthermore, after revising the report by Khalil et al. [28], it seems that the excess in mortality described was attributable to the patients from the COVID-19 registry of Brazil, which shows an HDI belonging to group 2 [13]. While currently few results have been described for countries with a HDI lower than group 1, such as Iran [40], which is also encompassed in group 2 [13], it should be considered that in these countries, there is likely a higher proportion of obesity, diabetes, cardiovascular disease, and other maternal comorbidities and obstetric morbidities, which could contribute to a poor prognosis in cases of SARS-CoV-2 coinfection. As the HDI comprises information on average life expectancy, adherence to the health system, and the education and economic level of each population, this indicator has been proposed as a variable to consider when analyzing MPMM [18].

Our findings indicate the importance of classifying maternal origin and social status to understand the risks suffered by immigrant pregnant women as vulnerable groups in Spain and countries with a similar HDI such as Italy, UK, France or the USA [35].

While a lower country of origin HDI is a predictive factor of a worse maternal–perinatal prognosis, we observed that this risk was not modified by the presence of other factors such as overweight, obesity, preexisting maternal comorbidity, and obstetric comorbidity. Thus, patients from a country with an HDI lower than group 1 showed a three-fold significantly higher likelihood of presenting with a maternal COVID-19-related morbidity event. Although it is not possible to act on maternal origin, it is important that clinicians consider the habits and customs of these patients as a preventive target of a poor maternal–perinatal prognosis. We believe that maternal country HDI in itself could be used to understand the effect of maternal origin on pregnancy outcomes [18,35]. Analysis of all these factors is the key to minimize the risk of loss of maternal–perinatal well-being, which could have a marked impact on the health of pregnant women and their newborns.

As a strength of our study, we should mention the large number of pregnant women with SARS-CoV-2 infection included of 1347 cases, which were recruited on a national level and matched with confirmed COVID-free peers in terms of delivery date. Moreover, ours is the first study to address the importance of characterizing SARS-CoV-2 infected pregnancies by maternal country of origin and assess the relationship of this sociodemographic index with adverse maternal–perinatal morbidity–mortality events.

Among the limitations of this study, missing data for some variables could have been as high as 30%. Moreover, country of origin was not established in 16% of the patients, reducing the accuracy and validity of our results.

The data compiled for this study offer information on the economic status, education level, and life expectancy of pregnant women, as these are components of the HDI. However, HDI simplifies and captures only part of what human development entails and does not reflect on inequalities, which could be important and should also be assessed in future studies. Moreover, as the health dimension of HDI itself is assessed by life expectancy at birth, it could be argued that pregnant women from low HDI countries would have higher maternal–perinatal morbidity as a starting point.

Finally, knowing that patients from countries with a lower HDI show a greater risk of morbidity and mortality due to COVID-19, these factors should be considered along with obesity, diabetes, or obstetric factors such as preeclampsia. Our findings provide direction for future studies designed to confirm the idea that maternal country of origin modifies SARS-CoV-2 infection prognosis in pregnant women. We additionally encourage to stress that being knowledgeable and having a decent standard of living (two HDI elements) are crucial factors for public health and SARS-CoV-2 infection outcomes.

## 5. Conclusions

SARS-CoV-2 infection increases the risk of maternal and perinatal morbidity and mortality both individually and jointly. There were more pregnant women from countries with a lower HDI than corresponding to group 1 among our cases than controls. These women also showed a greater risk of both maternal and perinatal morbidity and mortality.

Compared to other risk factors such as overweight, obesity, preexisting and obstetric comorbidities, HDI emerged as an independent risk factor explaining much of the increased maternal–perinatal morbidity and mortality detected in our group of cases. Further work is needed to confirm the real impact of this factor and its components on pregnancy outcomes.

## Figures and Tables

**Table 1:** Maternal characteristics.

	Number (%) of Patients Reporting Results	Overall	Cases	Controls	*p* Value
*n,* %	2694 (100)	2694 (100)	1347 (50)	1347 (50)	
**Maternal characteristics**					
Maternal age, mean, CI 95%	2664 (98.9)	32.6 (32.4–32.9)	32.7 (32.3–33.0)	32.6 (32.3–32.9)	0.734
Tobacco, *n,* %	2554 (94.8)	293 (11.5)	131 (10.2)	162 (12.8)	0.035
BMI, mean, CI 95%	2314 (85.9)	26.5 (26.3–26.8)	26.6 (26.3–26.9)	26.5 (26.2–26.8)	0.532
Maternal morbidities, *n,* %	2432 (90.3)	893 (36.7)	483 (39.2)	410 (34.1)	0.009
Nuliparous, *n,* %	2670 (99.1)	1059 (39.7)	516 (48.7)	543 (51.3)	0.315
Symptomps at triage, *n,* %	1457 (54.1)	644 (44.2)	632 (55.0)	12 (3.9)	<0.001
Pneumonia, *n,* %	1346 (50.0)	124 (9.2)	124 (12.2)	0 (0.0)	<0.001
**Obstetric and perinatal characteristics**					
Obstetric morbidities, *n,* %	2246 (83.4)	879 (39.1)	468 (42.5)	411 (35.9)	0.001
GA at triage, mean, CI 95%	2691 (99.9)	36.9 (36.7–37.1)	34.9 (34.5–35.3)	38.9 (38.8–39.0)	<0.001
GA at delivery, mean, CI 95 %	2694 (100)	38.8 (38.7–38.9)	38.5 (38.4–38.7)	39.0 (38.9–39.1)	<0.001
Birthweight, mean, CI 95%	2659 (98.7)	3219.5 (3198.2–3240.8)	3181.2 (3148.6–3213.7)	3257.7 (3230.3–3285.1)	<0.001
**Maternal and perinatal mortality**					
Maternal mortality, *n,* %	2694 (100)	2 (0.1)	2 (0.1)	0 (0)	0.096
Perinatal mortality, *n,* %	2694 (100)	21 (0.8)	16 (1.2)	5 (0.4)	<0.001
**Overall Maternal Morbidity**	1930 (71.6)	741 (38.4)	438 (41.3)	303 (34.8)	0.003
**COVID-19 Maternal Morbidity**	2694 (100)	96 (3.6)	93 (6.9)	3 (0.2)	<0.001
Oxygen therapy, *n,* %	2694 (100)	74 (2.8)	73 (5.4)	1 (0.1)	<0.001
Mechanical ventilation, *n,* %	2694 (100)	17 (0.6)	17 (1.3)	0 (0)	<0.001
Admission to ICU, *n,* %	2694 (100)	38 (1.4)	36 (2.7)	2 (0.2)	<0.001
**Not COVID-19 Maternal Morbidity**	1925 (71.5)	707 (36.7)	405 (38.4)	302 (34.7)	0.096
C-section, *n,* %	2687 (99.7)	640 (23.8)	373 (27.8)	267 (19.9)	<0.001
Haemorrhagic maternal disorders, *n,* %	1741 (64.6)	81 (4.7)	43 (4.4)	38 (5.0)	0.582
Hypertensive maternal disorders, *n,* %	2157 (80.1)	58 (2.7)	40 (3.6)	18 (1.7)	0.006
**Perinatal morbidity**	2165 (80.4)	313 (14.5)	211 (19.0)	102 (9.7)	<0.001
GA< 37 weeks, *n,* %	2694 (100)	230 (8.5)	149 (11.1)	81 (6.0)	<0.001
Neonatal Near miss, *n,* %	2642 (98.1)	90 (3.4)	65 (4.9)	25 (1.9)	<0.001
GA < 33 weeks, *n,* %	2694 (100)	68 (2.5)	53 (3.9)	15 (1.1)	<0.001
Birthweight < 1750 grs, *n,* %	2659 (98.7)	47 (1.8)	34 (2.6)	13 (1.0)	0.002
Apgar 5 minutes < 7, *n,* %	2664 (98.9)	24 (0.9)	12 (0.9)	12 (0.9)	0.988
Admission to NICU, *n,* %	2694 (100)	166 (6.2)	137 (10.2)	29 (2.2)	<0.001

**Table 2:** Maternal and perinatal characteristics.

	Number (%) of Patients Reporting Results	High/Medium/Low HDI	Very High HDI	*p* Value
*n,* %	2263 (84.0)	696 (30.8)	1567 (69.2)	
Cases, *n,* %	2263 (84.0)	456 (65.5)	678 (43.3)	<0.001
Control cases, *n,* %	2263 (84.0)	240 (34.5)	889 (56.7)	<0.001
**Maternal and perinatal mortality**				
Maternal mortality, *n,* %	1549 (57.5)	1 (0.2)	1 (0.1)	0.565
Perinatal mortality, *n,* %	2263 (84.0)	7 (1.0)	10 (0.6)	0.362
**Overall Maternal Morbidity**	1659 (61.6)	211 (39.4)	423 (37.6)	0.480
**COVID-19 Maternal Morbidity**	2263 (84.0)	42 (6.0)	38 (2.4)	<0.001
Oxygen therapy, *n,* %	2263 (84.0)	33 (4.7)	28 (1.8)	<0.001
Mechanical ventilation, *n,* %	2263 (84.0)	8 (1.2)	9 (0.6)	0.158
Admission to ICU, *n,* %	2263 (84.0)	18 (2.6)	16 (1.0)	0.007
**Not COVID-19 Maternal Morbidity**	1656 (61.5)	196 (36.7)	411 (36.6)	0.977
C-section, *n,* %	2258 (83.8)	172 (24.8)	377 (24.1)	0.729
Haemorrhagic maternal disorders, *n,* %	1499 (55.6)	27 (5.5)	45 (4.5)	0.364
Hypertensive maternal disorders, *n,* %	1841 (68.3)	22 (3.8)	30 (2.4)	0.106
**Perinatal morbidity**	1801 (66.9)	84 (14.6)	179 (14.6)	0.996
GA< 37 weeks, *n,* %	2263 (84.0)	56 (8.1)	143 (9.1)	0.399
Neonatal Near miss, *n,* %	1768 (65.6)	28 (4.9)	45 (3.8)	0.251
GA < 33 weeks, *n,* %	2263 (84.0)	23 (3.3)	38 (2.4)	0.242
Birthweight < 1750 grs, *n,* %	2237 (83.0)	16 (2.3)	27 (1.8)	0.369
Apgar 5 minutes < 7, *n,* %	2239 (83.1)	5 (0.7)	13 (0.8)	0.786
Admission to NICU, *n,* %	2263 (84.0)	52 (7.5)	84 (5.4)	0.056

## Data Availability

The data used to support the findings of the present study are available from the corresponding author upon request.
